# Low skeletal muscle mass and treatment outcomes among adults with haematologic malignancies: A systematic review and meta‐analysis

**DOI:** 10.1002/jcsm.13446

**Published:** 2024-04-01

**Authors:** Nadia M. Anabtawi, Monica Sai Pasala, Alyssa A. Grimshaw, Prakash Kharel, Susan Bal, Kelly Godby, Ashmita Siwakoti, Thomas W. Buford, Smita Bhatia, Luciano J. Costa, Grant R. Williams, Smith Giri

**Affiliations:** ^1^ School of Medicine University of Alabama at Birmingham Birmingham AL USA; ^2^ Harvey Cushing/John Hay Whitney Medical Library Yale University New Haven CT USA; ^3^ Department of Hospital Medicine Geisinger Health System, Geisinger Danville PA USA; ^4^ Department of Medicine, Division of Hematology and Oncology University of Alabama at Birmingham Birmingham AL USA; ^5^ Department of Medicine University of Kentucky Lenxtington KY USA; ^6^ Department of Medicine, Division of Gerontology, Geriatrics & Palliative Care University of Alabama at Birmingham Birmingham AL USA; ^7^ Birmingham/Atlanta VA GRECC Birmingham VA Medical Center Birmingham AL USA; ^8^ Institute for Cancer Outcomes and Survivorship University of Alabama at Birmingham Birmingham AL USA

**Keywords:** Adverse outcomes, Muscle mass, Prognostication, Sarcopenia, Toxicities

## Abstract

**Background:**

Low skeletal muscle mass (LSMM) and/or, function associated with an increased risk of treatment‐related toxicities and inferior overall survival (OS) among adults with solid malignancies. However, the association between LSMM and treatment‐related toxicities among adults with haematologic malignancies remains unclear.

**Methods:**

Using a pre‐published protocol (CRD42020197814), we searched seven bibliographic databases from inception to 08/2021 for studies reporting the impact of LSMM among adults ≥18 years with a known haematologic malignancy. The primary outcome of interest was OS, and secondary outcomes included progression free survival (PFS) and non‐relapse mortality (NRM). These effect sizes were quantified in terms of hazards ratio (HR) along with 95% confidence interval (CI) and pooled across studies using a DerSimonian–Laird random‐effects model. Heterogeneity was assessed using the Cochran's *Q* and the *I* ^2^ statistic. All hypothesis testing was two‐sided with an alpha of 0.05.

**Results:**

Of 3791 studies screened, we identified 20 studies involving 3468 patients with a mean age of 60 years; 44% were female and the most common malignancy was diffuse large B‐cell lymphoma (42%). Most studies measured muscle mass using single slice computed tomography imaging at the L3 level. The presence of LSMM was associated with worse OS (pooled HR = 1.81, 95% CI = 1.48–2.22, *P* < 0.001) with moderate heterogeneity (Cochran's *Q*, *I* ^2^ = 60.4%), PFS (pooled HR = 1.61, 95% CI = 1.28–2.02, *P* < 0.001) with moderate heterogeneity (Cochran's *Q*, *I* ^2^ = 66.0%). Similarly, LSMM was associated with worse NRM (HR = 1.72, 95% CI = 1.34–2.22, *P* < 0.001) with little evidence of heterogeneity (Cochran's *Q*, *I* ^2^ = 0.0%).

**Conclusions:**

LSMM is associated with worse survival outcomes among adults with haematologic malignancies. Further research into understanding the underlying mechanism of this association and mitigating the negative effects of LSMM among adults with haematologic malignancies is needed.

## Introduction

Sarcopenia, coined by Irwin Rosenberg in 1988, is defined as a *syndrome characterized by progressive and generalized loss of skeletal muscle mass (SMM) and strength, associated with adverse outcomes like physical disability, poor quality of life, and death*.^1^ Age‐related muscle mass decline can begin as early as the fourth decade of life and continues linearly with age.^2^ Sarcopenia's clinical significance in older adults has garnered attention over the past 3 decades with multiple studies associating it with increased likelihood of adverse outcomes including falls, fractures, physical disability and all‐cause mortality.^2^, ^3^

Despite longstanding knowledge of sarcopenia and its prognostic value in the general population, its significance among cancer patients was not realized until more recently. In 2004, Shen et al. reported that a single cross‐sectional imaging at the lumbar vertebral level can be used to accurately measure total body muscle mass.^4^ Mourtzakis et al. later validated this method among cancer patients using computed tomography images acquired during routine cancer care.^5^ Since then, a number of authors have studied the impact of low skeletal muscle mass (LSMM) in cancer populations within a relatively short period, making it necessary to perform a comprehensive review to summarize the collective evidence to date on the clinical significance of sarcopenia among patients with cancer. To that end, systematic reviews on the impact of sarcopenia among patients with solid tumours have been published.^6^ These studies demonstrate that the presence of LSMM correlates with worse survival in individuals diagnosed with solid tumours.^6^ However, to our knowledge, only few older studies have focused among patients with haematologic malignancies.^7^, ^8^ Given the varying demographic characteristics, treatment regimens and life expectancies of patients with haematologic malignancies versus solid tumours, a separate and updated review on the impact of LSMM on adverse outcomes in this population is warranted.

In this systematic review and meta‐analysis, we summarize the evidence to date on the prevalence and impact of LSMM on adverse outcomes among adults with haematologic malignancies.

## Methods

This systematic review and meta‐analysis was performed in accordance with a previously published protocol (CRD42020197814). Our findings are reported according to the Preferred Reporting Items for Systematic Reviews and Meta‐Analysis (PRISMA) statement for meta‐analysis.^9^ The checklist is provided in Table S1.

### Search strategy and selection criteria

A systematic search of the literature was conducted by a medical librarian (AAG) in Cochrane Library, Google Scholar, Ovid Embase, Ovid MEDLINE, PubMed, Scopus, and Web of Science Core Collection databases to identify relevant articles published from the inception of each database to August 16, 2022. The search was peer‐reviewed by a second medical librarian using PRESS (Peer Review of Electronic Search Strategies).^10^ Databases were searched using a combination of controlled vocabulary and free text terms for sarcopenia, SMM, and haematologic malignancies. The search was not limited by publication type or year. Search strategies for all databases used in this study can be found in Table S2. CitationChaser was used to search the reference lists of included studies to find additional relevant studies not retrieved by the database search.^11^

### Selection criteria

After preliminary screening, the full text of potentially eligible studies was reviewed independently by two authors (N. A. and M. S. P.) to confirm final eligibility for qualitative and quantitative synthesis based on the following inclusion criteria: (a) studies evaluating muscle mass with or without muscle function among adults ≥18 years diagnosed with a haematologic malignancy (as defined by the World Health Organization); (b) involving measurements of muscle mass using a validated tool including dual‐energy X‐ray absorptiometry (DXA), computed tomography, magnetic resonance imaging, or bioelectrical impedance analysis (BIA); and (c) reporting the impact of LSMM on at least one clinical outcome. Relevant clinical outcomes of interest included at least one of the following: progression‐free survival (PFS), overall (OS) or cancer‐specific survival and/or treatment related toxicity/adverse events. The exclusion criteria were (a) studies of patients with non‐haematologic malignancy, or a combination of both solid tumours and haematologic malignancies that did not report outcomes separately for haematologic malignancies; (b) studies published in an abstract form only; (c) review articles, editorials, commentaries or basic research articles; (d) case reports or case series with <5 patients; (e) duplicate publications from the same patient cohort; (f) clinical trials without published results; and (g) studies without availability of full text in English.

### Data extraction

Two authors (N. A. and M. S. P.) independently reviewed the final eligible studies and extracted data sheet using the Covidence extraction tool for the study characteristics, which included the types of studies, number of participants, malignancies included, and methods used to define and measure LSMM. A third author (S. G.) included or excluded the studies that required consensus by reviewing the study and utilizing the study characteristics noted by the original two authors (N. A. and M. S. P.). The subset of articles included in the study was agreed upon by all authors. A separate pre‐piloted excel spreadsheet was used to extract the final clinical outcome data, which included the effect sizes for OS, PFS, and non‐relapse mortality (N. R. M.).

### Definition of low skeletal muscle mass

For this systematic review, we allowed studies that defined LSMM based on measurement of muscle mass regardless of muscle function/performance.^1^ Given the lack of a universally agreed‐upon cut‐point for computed tomography (CT) measures of muscle mass below, which someone would be considered to be sarcopenic, we followed the sarcopenia cut‐point definitions used by individual studies.^1^

### Definition of clinical outcomes

The primary efficacy outcomes included overall survival (OS), defined as the length of time from the date of diagnosis or start of treatment of the malignancy to date of death or last contact if alive; progression‐free survival (PFS), defined as time from clinical trial random assignment to the date of first‐confirmed progression or date of death, whichever happened first; and NRM, defined as death without relapse/recurrence after treatment. We quantified the effect measure in terms of hazard ratio (HR) along with 95% confidence interval (CI). If multiple publications were available from the same study, the one with the longest available follow‐up results was used to extract the summary effect.

### Quality assessment

We used the Joanna Briggs Institute (JBI) Critical Appraisal Tool to assess the quality of the 20 published studies included in this systematic review.^12^ By following the JBI's 11‐question checklist for cohort studies, two co‐authors (N. A. and M. S. P.) independently conducted a critical appraisal of the included studies to determine the quality and extent of bias in study design and analysis. The 11 categories address the following domains: method of recruitment and selection bias; consistency in the measurement of SMM; identification of confounding variables and whether they were accounted for through multivariable analyses; the validity and reliability of the measurements of SMM and outcomes, including a clear explanation of how death was determined; a follow‐up time point of at least 1 year with an event rate of >10%; identification of significant losses to follow‐up and whether they were accounted for through Kaplan–Meier or Cox regression analysis. Consensus was reached via discussion among authors about any discrepancies and each study was then given an overall appraisal score that conveyed inclusion or exclusion, with a plan to exclude any study that had three or more ‘no’ or ‘unclear’ quality categories.

### Statistical analysis

All statistical analyses were conducted according to a pre‐published protocol. We extracted the study level baseline characteristics (such as age, sex, and cancer type) to estimate pooled summary measures. For studies that did not report continuous variables in terms of means and standard deviations, we estimated these measures using median, range and sample size using a validated method suggested by Hozo et al.^13^ We extracted study level OS, PFS, and NRM HRs along with 95% CI comparing patients with LSMM versus patients with normal SMM as the reference category. Subsequently, we pooled relative log HRs across multiple studies using the DerSimonian–Laird random effects mode. We chose to pool effect size using random effects model, *a priori*, as we anticipated the eligible studies to include heterogeneous populations with varying treatment regimens. However, to assess the robustness of our overall pooled estimates, we used alternative pooling methods including fixed effects model, Henmi–Copas approximate Gamma model, profile likelihood with Bartlett's correction, Doi's inverse variance heterogeneity model, restricted maximum likelihood estimation using Kenward and Roger's approximate correction to the covariance matrix and degrees of freedom. We assessed study level heterogeneity using Cochran's *Q* and *I* ^2^ statistic and explored evidence of any substantial heterogeneity with appropriate sensitivity/subgroup analysis. We conducted subgroup analyses for patients with the three major categories of haematologic malignancies, that is, leukaemia, lymphoma, and multiple myeloma. To identify potentially influential studies, we conducted a leave‐one‐out meta‐analysis where we computed overall effect size after removing one study at a time. Lastly, we evaluated for the presence of publication bias using funnel plot and Egger's regression intercept. All statistical analyses were performed using admetan and meta package on STATA v16.0 (StataCorp LLC College Station Tx). All *P* values were two‐sided and the level of significance was set at 0.05.

## Results

Our systematic search yielded 7095 citations (Figure S5). After removing duplicates, 4547 citations underwent title and abstract screening, and 143 citations met the criteria for full text review. Of these, 126 papers were excluded for no outcomes data, ineligible patient population, ineligible outcomes, conference abstracts, duplicate study data, non‐English language, no original data, ineligible comparator, duplicate, or ineligible settings (Table S3). Twenty studies involving 3468 patients were selected for final qualitative and quantitative synthesis.

The pooled mean age of the patient population was 60 years and 44% were females. The most common malignancy was diffuse large B‐cell lymphoma (42%). The patients received various treatment regimens including combination chemoimmunotherapy, and autologous or allogeneic haematopoietic cell transplantation. Table 1 presents the characteristics of the study populations across these 20 studies.

**Table 1:** Summary of demographic and defining characteristics of the patients of the included clinical trials

Author (year)	Diagnosis	Treatment	No. of patients	Median age; years (range)	Female (%)	LSMM (%)	Estimation of sarcopenia	Endpoints
Ando (2020)	AML, MDS	Allo‐HCT	125	47 (16–65)	52 (41.6%)	52 (41.6%)	CT at L3 level	OS/PFS/NRM
Armenian (2019)	ALL, AML, and MDS	Allo‐HCT	859	51 (18–74)	395 (50.0%)	290 (33.8%)	CT at L3 level	OS/NRM
Armenian (2020)	HL and NHL	Allo‐HCT	320	53.3 (18.5–78.1)	122 (38.1%)	109 (34.1%)	CT at L3 level	OS
Besutti (2021)	DLBCL	R‐CHOP	116	63.7^a^	56 (48.3%)	NR	CT at L3 level	OS/PFS
Burkart (2019)	DLBCL, MCL, and BL	Chemotherapy	109	64 (44.8–74.8)	56 (51.4%)	65 (59.6%)	CT or PET/CT at L3 level	OS
Camus (2014)	DLBCL	R‐CHOP	80	78 (70–88)	45 (56.3%)	44 (55%)	CT at L3 level	OS/PFS
Chu (2017)	DLBCL	R‐CHOP	224	62 (21–88)	99 (44.2%)	NR	CT at L3 level	OS/PFS
Chu (2015)	FL	Chemotherapy	145	57 (29–83)	65 (44.8%)	NR	CT at L3 level	OS
Go (2016)	DLBCL	R‐CHOP	187	61.6	75 (40.1%)	46 (24.6%)	CT at T4 level	OS/PFS
Iltar (2021)	DLBCL	R‐CHOP	120	59.1 (52–68)	54 (45.0%)	65 (54.2%)	CT at psoas level	OS/PFS
Jung (2021)	AML	Chemotherapy, HMA	96	58 (18–84)	12 (12.5%)	37 (38.5%)	CT or PET/CT at L1 level	OS/PFS
Lanic (2014)	DLBCL	R‐CHOP	83	78 (70–95)^a^	46 (56.1%)	45 (54.9%)	CT at L3 level	OS/PFS
Lin (2020)	HL and NHL	Allo‐HCT	146	NR	NR	80 (54.8%)	CT at L3 level	OS/PFS
Nakamura (2015)	DLBCL	R‐CHOP	207	67 (19–86)	86 (41.5%)	115 (55.6%)	CT at L3 level	OS/PFS
Nakamura (2019)	AML	Chemotherapy, HCT	90	59 (18–84)	39 (43.3%)	39 (43.3%)	CT at L3 level	OS/PFS
Rier (2020)	DLBCL	R‐CHOP	164	64.5 (54.3–74)	NR	NR	CT at L3 level	OS/PFS
Takeoka (2016)	MM	Bor/IMiD based, aPBSCT	56	71 (65–75)	37 (66.1%)	37 (66.1%)	CT or PET/CT at L3 level	OS
Williams (2020)	MM	Auto‐HCT	142	62.4 (38.2–78.7)	50 (35.2%)	72 (50.7%)	CT at L3 level	OS/PFS
Zakaria (2018)	MM‐SM	SBRT	46	64.8^a^	15 (32.6%)	NR	CT at L4 level	OS
Zilioli (2021)	HL	Any	154	71^a^	76 (49.4%)	113 (73.4%)	CT or PET/CT at L3 level	OS/PFS

^a^ Mean ages provided only.

chemotherapy, not specified which type; HMA, hypomethylating agents; LSMM, low skeletal muscle mass; NR, not reported; R‐CHOP, rituximab‐cyclophosphamide‐hydroxydaunorubicin‐oncovin‐prednisone; SBRT, stereotactic body radiation therapy.

### Quality of studies

Using the JBI tool, all 20 studies received an overall high‐quality rating. In the JBI tool for cohort studies, category seven assesses the validity of the survival outcomes, and we found that only two studies^14^, ^15^ clearly reported that survival was reported based on official national death records. Four studies^16^, ^17^, ^18^, ^19^ did not provide a statement on how death was reported, and this ambiguity introduced bias. Quality assessment of these studies is presented in Table 2.

**Table 2:** Quality assessment of included studies using the Joanna Briggs institute (JBI) critical appraisal checklist for cohort studies

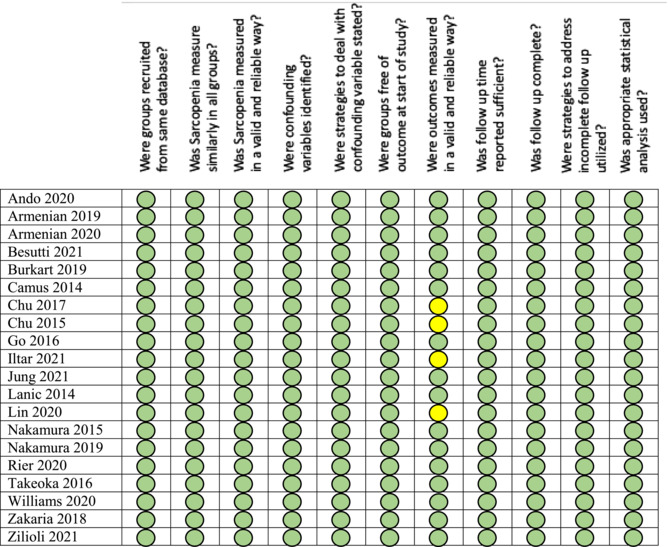

Green colour signifies yes to the quality assessment question. Yellow colour signifies unclear.

### Measurement of skeletal muscle mass

All 20 studies measured SMM by using CT images that were obtained as a part of routine oncologic care. Overall, 80% of the studies^14^, ^15^, ^16^, ^17^, ^19^, ^20^, ^21^, ^22^, ^23^, ^24^, ^25^, ^26^, ^27^, ^28^, ^29^, ^30^ utilized cross‐sectional images at the level of L3 vertebral body to measure skeletal muscle area. Only 15%^31^, ^32^, ^33^ used muscle measurements at other vertebral body levels (T4, L1, and L4) and 5% used measurements at the psoas muscle level.^18^ None of the studies used objective tests of muscle function/performance, such as handgrip strength or gait speed, as recommended in the updated European Working Group on Sarcopenia in Older People (EWGSOP2) definition.^1^

### Cut‐points used to define low muscle mass

Of the 20 studies, 55% used pre‐existing sex‐specific cut‐points from prior literature to define LSMM in their patient population. Thirty per cent of studies^14^, ^15^, ^17^, ^19^, ^21^, ^28^ used standardized and validated cut‐offs by Martin 2013^34^ and Prado 2009,^35^ which identified optimum cut‐points of LSMM to be <43 cm/m^2^ (BMI < 25 kg/m^2^) or <53 cm/m^2^ (BMI ≥ 25 kg/m^2^) in males and <41 cm/m^2^ (regardless of BMI) in females. Ten per cent of the studies^20^, ^26^ used the pre‐existing definition of LSMM by Lanic 2013^25^ which was <55cm^2^/m^2^ for males and <39 cm^2^/m^2^ for females, while another 10%^22^, ^27^ used pre‐existing cut‐points from other literature. Five per cent^30^ of the studies utilized cut‐points that were defined based on pre‐existing census on cancer cachexia.

The remaining 45% of the studies in this review developed internal data‐driven cut‐points, with 10%^16^, ^25^ using the minimum *P* value method, 10%^31^, ^33^ categorizing the lowest quartile of SMI in their patient population to be sarcopenic, 5%^29^ categorizing patients with ≤80% high‐density muscle to be sarcopenic, and 20%^18^, ^23^, ^24^, ^32^ using receiver operator curve analysis to identify sex‐specific cut‐offs for their population's CT measurements.

### Impact of low skeletal muscle mass on overall survival

**Figure 1:**
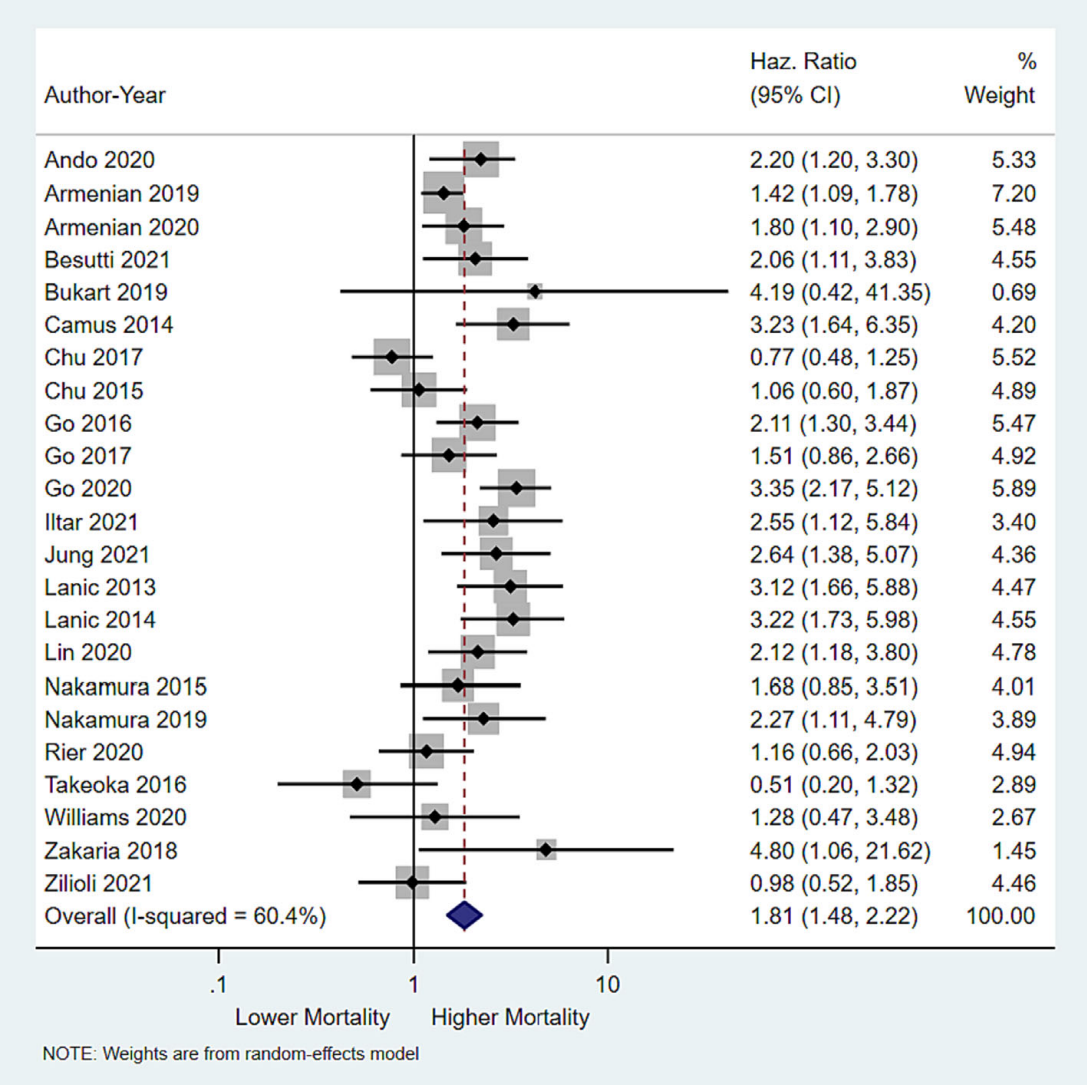
Forrest plot showing impact of low skeletal muscle mass on overall survival. As compared with those with normal skeletal muscle mass, patients with low skeletal muscle mass had worse overall survival (pooled hazard ratio 1.81; 95% CI 1.48–2.22), *P* value <0.001).

Of the 20 studies reporting on the impact of LSMM on all‐cause mortality, all but three studies^17^, ^20^, ^21^ had HR point estimates suggesting a detrimental impact of LSMM on survival. This was confirmed in our pooled analysis which showed that LSMM was associated with a significantly increased risk of all‐cause mortality (pooled HR = 1.81 (95% CI = 1.48–2.22, *P* value <0.001) (Figure 1). There was evidence of moderate heterogeneity among the studies (*I* ^2^ = 60.4%; Cochran's *Q P* value .003).

### Impact of low skeletal muscle mass on progression‐free survival

**Figure 2:**
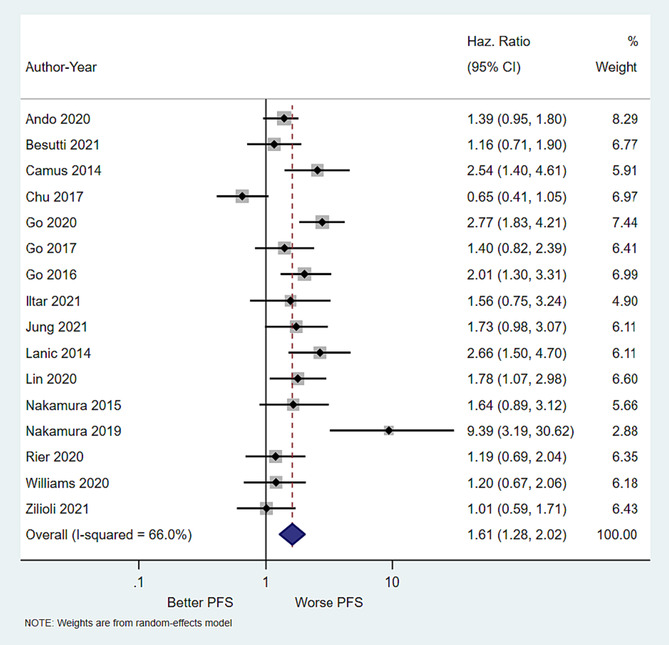
Forrest plot showing impact of low skeletal muscle mass on progression free survival. As compared with those with normal skeletal muscle mass, patients with low skeletal muscle mass had worse progression free survival (pooled hazard ratio 1.61; 95% CI 1.28–2.02), *P* value <0.001).

Overall, 13 studies reported PFS outcome data. Our meta‐analysis revealed that LSMM was associated with a significantly increased risk of progression or death (pooled HR = 1.61 (95% CI = 1.28–2.02, *P* value <0.001) (Figure 2). Again, there was evidence of moderate heterogeneity (*I* ^2^ statistic = 66.0%; Cochran's *Q P* value 0.02).

### Impact of low skeletal muscle mass on non‐relapse mortality

**Figure 3:**
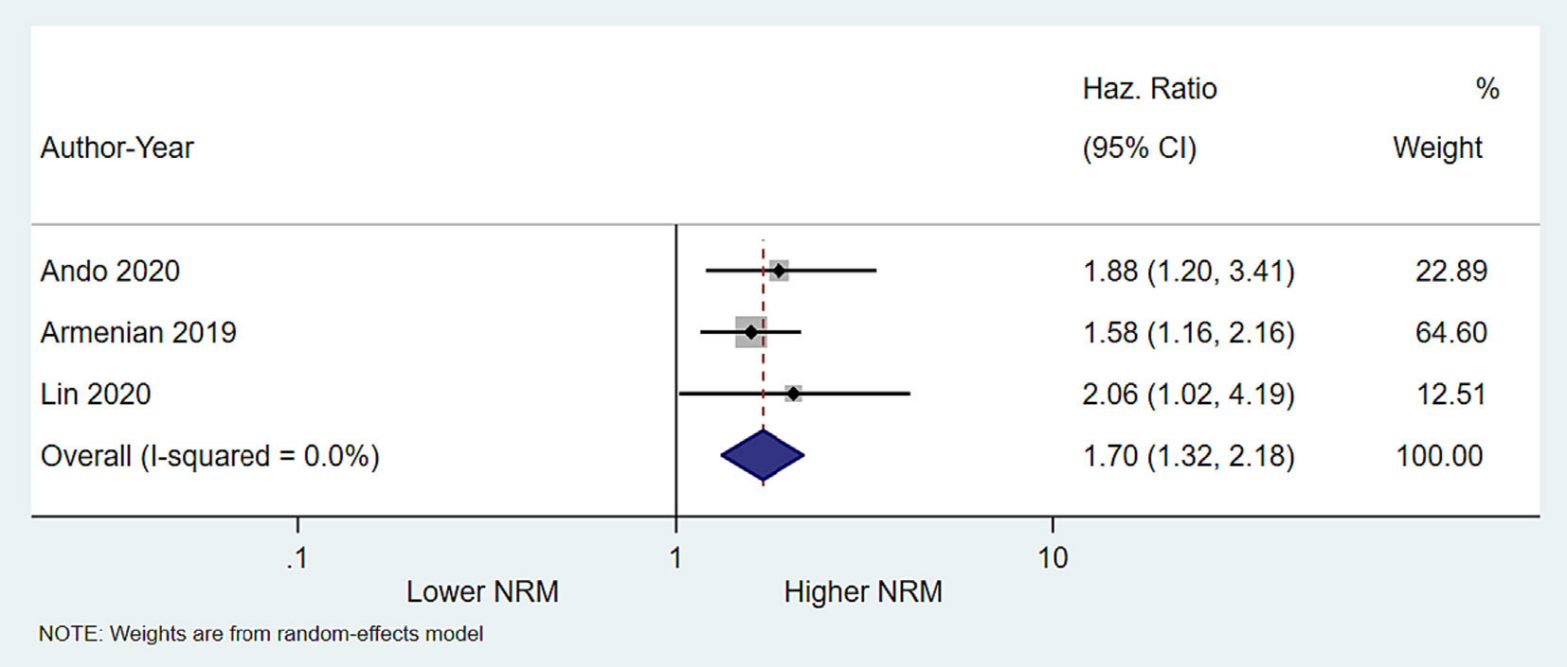
Forrest plot showing impact of low skeletal muscle mass on non‐relapse mortality. As compared with those with normal skeletal muscle mass, patients with low skeletal muscle mass had worse overall survival (pooled hazard ratio 1.72; 95% CI 1.34–2.22), *P* value <0.001).

Three studies in allogenic stem cell transplant recipients reported NRM as an outcome data. LSMM was associated with an increased risk of NRM (pooled HR = 1.72; 95% CI = 1.34–2.22, *P* < 0.001) with no evidence of heterogeneity (*I* ^2^ 0%; Cochran's *Q P* value 0.73) (Figure 3).

### Evaluation of publication bias

We evaluated the presence of publication bias or small study effects, visually by the use of funnel plots and statistically using Egger's regression intercept. Visually we found a largely symmetric appearing funnel plot (supporting information) showing no graphical evidence of small study effects; this was confirmed quantitatively by Egger's regression test, where the regression intercept was not significantly different from zero (β = 0.93; 95% CI −0.63 to 2.49, *P* value 0.26).

### Sensitivity and subgroup analysis

To further explore the moderate level of heterogeneity seen in our key results, we conducted a sub‐group analysis based on cancer type (myeloid, lymphoid, and other). Our results showed a largely consistent relationship in terms of effect size point estimates and direction on the impact of the LSMM on outcomes regardless of malignancy type (supporting information), albeit not statistically significant in the myeloma subgroup. To assess the robustness of our findings, we used alternative methods to estimate between study heterogeneity (***T*** ^2^), and our results were consistent regardless of the pooling method (supporting information). Lastly, we found no potential outliers with stable pooled hazard ratio estimates using a leave‐one‐out meta‐analysis approach (supporting information).

## Discussion

The findings of this systematic review and meta‐analysis suggest that the presence of LSMM in adult patients with haematologic malignancies is associated with significantly worse overall survival, progression‐free survival, and non‐relapse mortality. This adverse prognostic impact of LSMM appears to be consistent regardless of the type of haematologic malignancy.

The results of this study highlight the importance of LSMM in predicting adverse outcomes among patients with haematologic malignancies. Whereas these findings are consistent with the broader literature in the non‐cancer population,^2^, ^6^ the consequences are likely much greater among patients with cancer due to a high prevalence of LSMM as reported before,^36^, ^37^ and also seen in our study population (reported prevalence ranging from 24% to 73%). Prior to our study, two systematic reviews have been conducted to summarize the existant literature on the impact of LSMM on clinical outcomes among patients with haematologic malignancies.^7^, ^8^ Jia et al. summarized seven studies reporting on the association between sarcopenia and clinical outcomes among patients receiving haematopoietic stem cell transplantation and concluded that sarcopenia, defined as LSMM alone, was associated with 2 times higher odds of NRM and as well shorter OS.^8^ Meanwhile, Surov et al. reported that LSMM was associated with a 2 times increased risk of all‐cause mortality among patients with haematologic malignancies.^7^ Whereas our findings are in agreement with the above two studies, we have presented a more updated and rigorous analysis of 20 individual studies to comprehensively review the literature to date.

We observed that CT scans were uniformly used to quantify muscle mass in all 20 included studies. This is not surprising because most cancer patients undergo CT imaging for diagnosis, treatment planning, or follow up providing a unique opportunity to quantify muscle mass in these patients using well established methods.^4^, ^5^ Additionally, unlike traditional methods such as BIA, or DXA, CT images provide direct quantification of muscle mass and are therefore less prone to measurement errors.^38^ We believe that the resulting uniformity in measurement of muscle mass may have resulted in an improved overall quality and validity of our analysis. Yet we observed significant variation in the use of cut‐points for defining low muscle mass based on CT. It is worth noting that consensus groups such as EWGSOP have proposed cut‐points for identifying low muscle mass using DXA or BIA, but none for CT measurements.^3^ In our study, we found that the cut‐points provided by Prado et al.^39^ and Martin et al.^34^ were the most commonly employed, but there was significant variability with many studies using internal data driven cut‐points. We believe that such inconsistencies may have resulted in high level of heterogeneity observed in our pooled estimates.

Furthermore, we found that the existing literature on the relevance of sarcopenia in oncology often does not include a measurement of muscle function and instead only defines sarcopenia relative to muscle mass measurements. This is in sharp contrast to the newly updated EWGSOP2 criteria that emphasizes muscle strength/performance as a critical component of sarcopenia.^1^ This reductive, simplified definition ignores the multi‐faceted complexity of sarcopenia and does not fully convey the breadth of its clinical impact among patients with cancer. A comprehensive evaluation of the clinical impact of sarcopenia among patients with cancer should incorporate a measurement of muscle function, utilizing techniques such as a dynamometer to assess hang grip strength.

The strengths of our systematic review include the narrow focus of our research question with a unique patient population as well as our clearly defined criteria‐based selection of relevant studies. Our selected studies were critically appraised and concluded to be of high quality and validity. The uniform methodological approach of our systematic review objectively and comprehensively summarizes the current literature to reduce researcher implicit bias. However, there were limitations in our systematic review as well. Firstly, in our screening and selection process, we excluded conference abstracts and only included published, peer‐reviewed papers; this may have introduced potential publication bias. Nevertheless, we made a conscious decision to exclude abstracts due to potential for incomplete outcome results and inability to comprehensive assess methodological rigour and study quality. Secondly, we were unable to quantitatively assess the impact of sarcopenia on treatment‐related toxicities due to lack of uniform reporting on this outcome. Whereas few studies noted the presence of unfavourable treatment‐related effects that potentially led to treatment discontinuation, these results were not numerically summarized and thus could not be analysed objectively. Therefore, the impact of sarcopenia on treatment‐related toxicities in patients with haematologic malignancies remains relatively unclear at this time and future studies are needed to clarify this further. Our study population is heterogeneous due to the inclusion of different subtypes of haematologic malignancies. Our pooled effect sizes for OS have significant heterogeneity and as such merely reflect an average impact of LSMM across diverse haematologic malignancies. The degree of impact of LSMM on treatment outcomes may vary by the haematologic malignancy subtype, as suggested in our subgroup analysis. Lastly, we cannot definitively conclude on a causal association between sarcopenia and survival outcomes largely due to uniformly retrospective nature of existing studies. Rigorous studies with prospective outcome ascertainment as well as studies evaluating the impact of sarcopenia reversal on such relevant outcomes may help clarify this further.

In summary, our systematic review and meta‐analysis uniquely delves into the current evidence on the impact of sarcopenia on haematologic malignancies and shows that sarcopenia is associated with unfavourable survival outcomes among adults with haematologic malignancies. These observations warrant future well designed prospective studies to fully evaluate causality and underlying mechanisms and may ultimately pave the way for mitigating interventions to eventually improve patient outcomes.

## Funding

Supported in part by the Walter B. Frommeyer Fellowship in Investigative Medicine at the University of Alabama at Birmingham and the National Cancer Institute of the National Institutes of Health (K08CA234225). The content is solely the responsibility of the authors and does not necessarily represent the official views of the National Institutes of Health.

## Conflict of interest statement

SG: Honoraria: CareVive, OncLive; Research Funding: Carevive Systems, Pack Health, Sanofi. SB: Consulting or Advisory Role: Adaptive Biosciences; Research Funding: Amyloid Foundation. LC: Honoraria: Amgen, BMS, Janssen, Karyopharm Therapeutics, Sanofi, Takeda; Consulting or Advisory Role: AbbVie, Amgen, Celgene, Karyopharm Therapeutics; Speakers' Bureau: Amgen, Sanofi; Research Funding: Janssen, Amgen, BMS. None of the other authors have relevant disclosures.

## Supporting information


**Table S1:** Reporting Guideline Checklists.
**Table S2:** Search Strategies.
**Table S3:** Excluded Studies Table.
