# Supplementary material for: Low skeletal muscle mass and treatment outcomes among adults with haematologic malignancies: A systematic review and meta‐analysis
Source: J Cachexia Sarcopenia Muscle. 2024 Apr 1;15(3):1084–93. doi: 10.1002/jcsm.13446 (PMC11154774; doi:10.1002/jcsm.13446)
Supplement: Supplementary file 1 — Table S1: Reporting Guideline Checklists. Table S2: Search Strategies. Table S3: Excluded Studies Table. [file JCSM-15-1084-s001.docx]

**SUPPLEMENTARY FILES**

**Contents**

| PRISMA Checklists | **28-32** |
| --- | --- |
| Search Strategies | **33-36** |
| PRISMA flow diagram for study selection process | **37** |
| Table of Excluded Studies with reasons for exclusion | **38-47** |
| Funnel plot showing graphical evaluation for potential publication bias | **48** |
| Forrest plot of the leave-one-out meta-analysis to detect influential/outlier studies | **49** |
| Forrest plot showing pooled effect size for overall survival using alternative pooling strategies | **50** |
| Forrest plot showing subgroup analysis of the impact of sarcopenia based on malignancy sub-type | **51** |

**Supplementary Table 1: Reporting Guideline Checklists**

PRISMA 2020 Main Checklist

| **Topic** | **No.** | **Item** | **Location where item is reported** |
| --- | --- | --- | --- |
| **TITLE** |  |  |  |
| **Title** | 1 | Identify the report as a systematic review. | LN1-2 |
| **ABSTRACT** |  |  |  |
| **Abstract** | 2 | See the PRISMA 2020 for Abstracts checklist | Suppl.1 |
| **INTRODUCTION** |  |  |  |
| **Rationale** | 3 | Describe the rationale for the review in the context of existing knowledge. | LN70-91 |
| **Objectives** | 4 | Provide an explicit statement of the objective(s) or question(s) the review addresses. | LN92-93 |
| **METHODS** |  |  |  |
| **Eligibility criteria** | 5 | Specify the inclusion and exclusion criteria for the review and how studies were grouped for the syntheses. | LN108-120 |
| **Information sources** | 6 | Specify all databases, registers, websites, organisations, reference lists and other sources searched or consulted to identify studies. Specify the date when each source was last searched or consulted. | LN100-103 |
| **Search strategy** | 7 | Present the full search strategies for all databases, registers and websites, including any filters and limits used. | LN104-106, Supp. Table 2 |
| **Selection process** | 8 | Specify the methods used to decide whether a study met the inclusion criteria of the review, including how many reviewers screened each record and each report retrieved, whether they worked independently, and if applicable, details of automation tools used in the process. | LN111-124 |
| **Data collection process** | 9 | Specify the methods used to collect data from reports, including how many reviewers collected data from each report, whether they worked independently, any processes for obtaining or confirming data from study investigators, and if applicable, details of automation tools used in the process. | LN127-132 |
| **Data items** | 10a | List and define all outcomes for which data were sought. Specify whether all results that were compatible with each outcome domain in each study were sought (e.g. for all measures, time points, analyses), and if not, the methods used to decide which results to collect. | LN127-132 |
|  | 10b | List and define all other variables for which data were sought (e.g. participant and intervention characteristics, funding sources). Describe any assumptions made about any missing or unclear information. | LN127-132 |
| **Study risk of bias assessment** | 11 | Specify the methods used to assess risk of bias in the included studies, including details of the tool(s) used, how many reviewers assessed each study and whether they worked independently, and if applicable, details of automation tools used in the process. | LN146-159 |
| **Effect measures** | 12 | Specify for each outcome the effect measure(s) (e.g. risk ratio, mean difference) used in the synthesis or presentation of results. | LN131-132 |
| **Synthesis methods** | 13a | Describe the processes used to decide which studies were eligible for each synthesis (e.g. tabulating the study intervention characteristics and comparing against the planned groups for each synthesis (item 5)). | LN162-183 |
|  | 13b | Describe any methods required to prepare the data for presentation or synthesis, such as handling of missing summary statistics, or data conversions. | LN162-183 |
|  | 13c | Describe any methods used to tabulate or visually display results of individual studies and syntheses. | LN162-183 |
|  | 13d | Describe any methods used to synthesize results and provide a rationale for the choice(s). If meta-analysis was performed, describe the model(s), method(s) to identify the presence and extent of statistical heterogeneity, and software package(s) used. | LN162-183 |
|  |  |  |  |
|  | 13e | Describe any methods used to explore possible causes of heterogeneity among study results (e.g. subgroup analysis, meta-regression). | LN175-176 |
|  | 13f | Describe any sensitivity analyses conducted to assess robustness of the synthesized results. | LN162-183 |
| **Reporting bias assessment** | 14 | Describe any methods used to assess risk of bias due to missing results in a synthesis (arising from reporting biases). | LN146-156 |
| **Certainty assessment** | 15 | Describe any methods used to assess certainty (or confidence) in the body of evidence for an outcome. | LN179-183 |
| **RESULTS** |  |  |  |
| **Study selection** | 16a | Describe the results of the search and selection process, from the number of records identified in the search to the number of studies included in the review, ideally using a flow diagram. | LN186-191 |
|  | 16b | Cite studies that might appear to meet the inclusion criteria, but which were excluded, and explain why they were excluded. | LN187-191 |
| **Study characteristics** | 17 | Cite each included study and present its characteristics. | LN192-196 |
| **Risk of bias in studies** | 18 | Present assessments of risk of bias for each included study. | LN198-202 |
| **Results of individual studies** | 19 | For all outcomes, present, for each study: (a) summary statistics for each group (where appropriate) and (b) an effect estimate and its precision (e.g. confidence/credible interval), ideally using structured tables or plots. | LN204-258 |
| **Results of syntheses** | 20a | For each synthesis, briefly summarise the characteristics and risk of bias among contributing studies. | LN204-258 |
|  | 20b | Present results of all statistical syntheses conducted. If meta-analysis was done, present for each the summary estimate and its precision (e.g. confidence/credible interval) and measures of statistical heterogeneity. If comparing groups, describe the direction of the effect. | LN204-258 |
|  | 20c | Present results of all investigations of possible causes of heterogeneity among study results. | LN251-258 |
|  | 20d | Present results of all sensitivity analyses conducted to assess the robustness of the synthesized results. | LN251-258 |
| **Reporting biases** | 21 | Present assessments of risk of bias due to missing results (arising from reporting biases) for each synthesis assessed. | LN244-248 |
| **Certainty of evidence** | 22 | Present assessments of certainty (or confidence) in the body of evidence for each outcome assessed. | LN251-258 |
| **DISCUSSION** |  |  |  |
| **Discussion** | 23a | Provide a general interpretation of the results in the context of other evidence. | LN260-289 |
|  | 23b | Discuss any limitations of the evidence included in the review. | LN290-294 |
|  | 23c | Discuss any limitations of the review processes used. | LN294-307 |
|  | 23d | Discuss implications of the results for practice, policy, and future research. | LN308-318 |
| **OTHER INFORMATION** |  |  |  |
| **Registration and protocol** | 24a | Provide registration information for the review, including register name and registration number, or state that the review was not registered. | LN95-96 |
|  | 24b | Indicate where the review protocol can be accessed, or state that a protocol was not prepared. | LN95-96 |
|  | 24c | Describe and explain any amendments to information provided at registration or in the protocol. | - |
| **Support** | 25 | Describe sources of financial or non-financial support for the review, and the role of the funders or sponsors in the review. | LN323-326 |
| **Competing interests** | 26 | Declare any competing interests of review authors. | LN327-328 |
| **Availability of data, code and other materials** | 27 | Report which of the following are publicly available and where they can be found: template data collection forms; data extracted from included studies; data used for all analyses; analytic code; any other materials used in the review. | Available upon request |

From: Page MJ, McKenzie JE, Bossuyt PM, Boutron I, Hoffmann TC, Mulrow CD, et al. The PRISMA 2020 statement: an updated guideline for reporting systematic reviews. MetaArXiv. 2020, September 14. DOI: 10.31222/osf.io/v7gm2. For more information, visit: www.prisma-statement.org

**PRISMA Abstract Checklist**

| **Topic** | **No.** | **Item** | **Reported?** |
| --- | --- | --- | --- |
| **TITLE** |  |  |  |
| **Title** | 1 | Identify the report as a systematic review. | Yes |
| **BACKGROUND** |  |  |  |
| **Objectives** | 2 | Provide an explicit statement of the main objective(s) or question(s) the review addresses. | Yes |
| **METHODS** |  |  |  |
| **Eligibility criteria** | 3 | Specify the inclusion and exclusion criteria for the review. | Yes |
| **Information sources** | 4 | Specify the information sources (e.g. databases, registers) used to identify studies and the date when each was last searched. | Yes |
| **Risk of bias** | 5 | Specify the methods used to assess risk of bias in the included studies. | Yes |
| **Synthesis of results** | 6 | Specify the methods used to present and synthesize results. | Yes |
| **RESULTS** |  |  |  |
| **Included studies** | 7 | Give the total number of included studies and participants and summarise relevant characteristics of studies. | Yes |
| **Synthesis of results** | 8 | Present results for main outcomes, preferably indicating the number of included studies and participants for each. If meta-analysis was done, report the summary estimate and confidence/credible interval. If comparing groups, indicate the direction of the effect (i.e. which group is favoured). | Yes |
| **DISCUSSION** |  |  |  |
| **Limitations of evidence** | 9 | Provide a brief summary of the limitations of the evidence included in the review (e.g. study risk of bias, inconsistency and imprecision). | Yes |
| **Interpretation** | 10 | Provide a general interpretation of the results and important implications. | Yes |
| **OTHER** |  |  |  |
| **Funding** | 11 | Specify the primary source of funding for the review. | Yes |
| **Registration** | 12 | Provide the register name and registration number. | Yes |

**Supplementary Table 2: Search Strategies**

**Ovid Embase**

1 exp Muscular Atrophy/

2 (sarcopenia* or myopenia* or dynapenia* or frailty or frail).tw,kw.

3 (muscl* adj1 (atroph* or wast* or loss* or mass* or function* or low or lowest or decreas* or strength*)).tw,kw.

4 1 or 2 or 3

5 exp hematologic malignancy/

6 ((hematolog* or hematopoiet* or blood or bone marrow or lymphoid or lymphoid tissue* or histiocytosis) adj1 (neoplasm* or cancer* or malignan* or neoplasia* or lesion* or sarcoma* or tumor* or tumour*)).tw,kw.

7 ((Plasma Cell* or Plasmacytic or Mast cell*) adj3 (tumor* or tumour* or malignan* or cancer* or dyscrasia* or neoplasm*)).tw,kw.

8 (lymphoma* or Germinoblastic Sarcoma* or Reticulolymphosarcoma* or Germinoblastoma* or Hodgkin* or Non Hodgkin* or Non-Hodgkin* or NonHodgkin* or Lymphogranuloma* or Immunoproliferative Small Intestinal Disease* or alpha-Chain Disease* or alpha chain disease* or Small Cleaved Cell Lymphoma* or Reticulosarcoma* or Burkitt* tumor* or burkitt* tumour* or Lymphomatoid Granulomatoses or Lymphomatoid Granulomatosis).tw,kw.

9 (aleukaemia* or aleukemia* or hemoblastoma* or leukaemia* or leukemia* or leucaemia* or leucemia* or leukemogenesis or leucemia*).tw,kw.

10 (myeloma* or myeloplaxoma* or plasmacytoma* or plasma cytoma* or Kahler* disease or mastocytoma*).tw,kw.

11 5 or 6 or 7 or 8 or 9 or 10

12 4 and 11

**Ovid Medline**

1 exp Muscular Atrophy/

2 (sarcopenia* or myopenia* or dynapenia* or frailty or frail).tw,kf.

3 (muscl* adj1 (atroph* or wast* or loss* or mass* or function* or low or lowest or decreas* or strength*)).tw,kf.

4 1 or 2 or 3

5 exp Hematologic Neoplasms/

6 ((hematolog* or hematopoiet* or blood or bone marrow or lymphoid or lymphoid tissue* or histiocytosis) adj1 (neoplasm* or cancer* or malignan* or neoplasia* or lesion* or sarcoma* or tumor* or tumour*)).tw,kf.

7 ((Plasma Cell* or Plasmacytic or Mast cell*) adj3 (tumor* or tumour* or malignan* or cancer* or dyscrasia* or neoplasm*)).tw,kf.

8 (lymphoma* or Germinoblastic Sarcoma* or Reticulolymphosarcoma* or Germinoblastoma* or Hodgkin* or Non Hodgkin* or Non-Hodgkin* or NonHodgkin* or Lymphogranuloma* or Immunoproliferative Small Intestinal Disease* or alpha-Chain Disease* or alpha chain disease* or Small Cleaved Cell Lymphoma* or Reticulosarcoma* or Burkitt* tumor* or burkitt* tumour* or Lymphomatoid Granulomatoses or Lymphomatoid Granulomatosis).tw,kf.

9 (aleukaemia* or aleukemia* or hemoblastoma* or leukaemia* or leukemia* or leucaemia* or leucemia* or leukemogenesis or leucemia*).tw,kf.

10 (myeloma* or myeloplaxoma* or plasmacytoma* or plasma cytoma* or Kahler* disease or mastocytoma*).tw,kf.

11 5 or 6 or 7 or 8 or 9 or 10

12 4 and 11

**Web of Science**

#1 TS=((hematolog* or hematopoiet* or blood or "bone marrow" or lymphoid or "lymphoid tissue*" or histiocytosis) near/1 (neoplasm* or cancer* or malignan* or neoplasia* or lesion* or sarcoma* or tumor* or tumour*)) OR TS=(("Plasma Cell*" or Plasmacytic or "Mast cell*") near/3 (tumor* or tumour* or malignan* or cancer* or dyscrasia* or neoplasm*)) OR TS=(lymphoma* or "Germinoblastic Sarcoma*" or Reticulolymphosarcoma* or Germinoblastoma* or Hodgkin* or "Non Hodgkin*" or Non-Hodgkin* or NonHodgkin* or Lymphogranuloma* or "Immunoproliferative Small Intestinal Disease*" or "alpha-Chain Disease*" or "alpha chain disease*" or "Small Cleaved Cell Lymphoma*" or Reticulosarcoma* or "Burkitt* tumor*" or "burkitt* tumour*" or "Lymphomatoid Granulomatoses" or "Lymphomatoid Granulomatosis") OR TS=(aleukaemia* or aleukemia* or hemoblastoma* or leukaemia* or leukemia* or leucaemia* or leucemia* or leukemogenesis or leucemia*) OR TS=(myeloma* or myeloplaxoma* or plasmacytoma* or "plasma cytoma*" or "Kahler* disease" or mastocytoma*)

#2 TS=(sarcopenia* or myopenia* or dynapenia* or frailty or frail) OR TS=(muscl* near/1 (atroph* or wast* or loss* or mass* or function* or low or lowest or decreas* or strength*))

#3 #1 and #2

**Scopus**

( TITLE-ABS-KEY ( ( hematolog* OR hematopoiet* OR blood OR "bone marrow" OR lymphoid OR "lymphoid tissue*" OR histiocytosis ) W/1 ( neoplasm* OR cancer* OR malignan* OR neoplasia* OR lesion* OR sarcoma* OR tumor* OR tumour* ) ) OR TITLE-ABS-KEY ( ( "Plasma Cell*" OR plasmacytic OR "Mast cell*" ) W/3 ( tumor* OR tumour* OR malignan* OR cancer* OR dyscrasia* OR neoplasm* ) ) OR TITLE-ABS-KEY ( lymphoma* OR "Germinoblastic Sarcoma*" OR reticulolymphosarcoma* OR germinoblastoma* OR hodgkin* OR "Non Hodgkin*" OR non-hodgkin* OR nonhodgkin* OR lymphogranuloma* OR "Immunoproliferative Small Intestinal Disease*" OR "alpha-Chain Disease*" OR "alpha chain disease*" OR "Small Cleaved Cell Lymphoma*" OR reticulosarcoma* OR "Burkitt* tumor*" OR "burkitt* tumour*" OR "Lymphomatoid Granulomatoses" OR "Lymphomatoid Granulomatosis" ) OR TITLE-ABS-KEY ( aleukaemia* OR aleukemia* OR hemoblastoma* OR leukaemia* OR leukemia* OR leucaemia* OR leucemia* OR leukemogenesis OR leucemia* ) OR TITLE-ABS-KEY ( myeloma* OR myeloplaxoma* OR plasmacytoma* OR "plasma cytoma*" OR "Kahler* disease" OR mastocytoma* ) ) AND ( TITLE-ABS-KEY ( sarcopenia* OR myopenia* OR dynapenia* OR frailty OR frail ) OR TITLE-ABS-KEY ( muscl* W/1 ( atroph* OR wast* OR loss* OR mass* OR function* OR low OR lowest OR decreas* OR strength* ) ) )

**Cochrane Library**

#1 ((hematolog* or hematopoiet* or blood or "bone marrow" or lymphoid or "lymphoid tissue*" or histiocytosis) near/1 (neoplasm* or cancer* or malignan* or neoplasia* or lesion* or sarcoma* or tumor* or tumour*)):ti,ab OR (("Plasma Cell*" or Plasmacytic or "Mast cell*") near/3 (tumor* or tumour* or malignan* or cancer* or dyscrasia* or neoplasm*)):ti,ab OR (lymphoma* or "Germinoblastic Sarcoma*" or Reticulolymphosarcoma* or Germinoblastoma* or Hodgkin* or "Non Hodgkin*" or Non-Hodgkin* or NonHodgkin* or Lymphogranuloma* or "Immunoproliferative Small Intestinal Disease*" or "alpha-Chain Disease*" or "alpha chain disease*" or "Small Cleaved Cell Lymphoma*" or Reticulosarcoma* or "Burkitt* tumor*" or "burkitt* tumour*" or "Lymphomatoid Granulomatoses" or "Lymphomatoid Granulomatosis") OR (aleukaemia* or aleukemia* or hemoblastoma* or leukaemia* or leukemia* or leucaemia* or leucemia* or leukemogenesis or leucemia*):ti,ab OR (myeloma* or myeloplaxoma* or plasmacytoma* or "plasma cytoma*" or "Kahler* disease" or mastocytoma*):ti,ab

#2 (sarcopenia* or myopenia* or dynapenia* or frailty or frail):ti,ab OR (muscl* near/1 (atroph* or wast* or loss* or mass* or function* or low or lowest or decreas* or strength*)):ti,ab

**PubMed**

((("Plasma Cell*"[Title/Abstract] tumor* or "Plasma Cell*"[Title/Abstract] tumour* or "Plasma Cell*"[Title/Abstract] malignan* or "Plasma Cell*"[Title/Abstract] cancer* or "Plasma Cell*"[Title/Abstract] dyscrasia* or "Plasma Cell*"[Title/Abstract] neoplasm* or Plasmacytic tumor* or Plasmacytic tumour* or Plasmacytic malignan* or Plasmacytic cancer* or Plasmacytic dyscrasia* or Plasmacytic neoplasm* or "Mast cell*"[Title/Abstract] tumor* or "Mast cell*"[Title/Abstract] tumour* or "Mast cell*"[Title/Abstract]malignan* or "Mast cell*"[Title/Abstract]cancer* or "Mast cell*"[Title/Abstract] dyscrasia* or "Mast cell*"[Title/Abstract] neoplasm*) OR (hematolog* neoplasm* [Title/Abstract] or hematopoiet* neoplasm*[Title/Abstract] or blood neoplasm* [Title/Abstract] or "bone marrow"neoplasm* [Title/Abstract] or lymphoid neoplasm* [Title/Abstract] or "lymphoid tissue*" neoplasm* [Title/Abstract] or histiocytosis neoplasm*[Title/Abstract] or hematolog* cancer* [Title/Abstract] or hematopoiet* cancer* [Title/Abstract] or blood cancer* [Title/Abstract] or "bone marrow" cancer* [Title/Abstract] or lymphoid cancer* [Title/Abstract] or "lymphoid tissue*" cancer* [Title/Abstract] or histiocytosis cancer* [Title/Abstract] or hematolog* malignan* [Title/Abstract] or hematopoiet* malignan*[Title/Abstract] or blood malignan* [Title/Abstract] or "bone marrow" malignan* [Title/Abstract] or lymphoid malignan* [Title/Abstract] or "lymphoid tissue*" malignan* [Title/Abstract] or histiocytosis malignan* [Title/Abstract] or hematolog* neoplasia* [Title/Abstract] or hematopoiet* neoplasia* [Title/Abstract] or blood neoplasia*[Title/Abstract] or "bone marrow" neoplasia* [Title/Abstract] or lymphoid neoplasia* [Title/Abstract] or "lymphoid tissue*"neoplasia* [Title/Abstract] or histiocytosis neoplasia* [Title/Abstract] or hematolog* lesion* [Title/Abstract] or hematopoiet* lesion* [Title/Abstract] or blood lesion* [Title/Abstract] or "bone marrow" lesion* [Title/Abstract] or lymphoid lesion*[Title/Abstract] or "lymphoid tissue*" lesion* [Title/Abstract] or histiocytosis lesion* [Title/Abstract] or hematolog* sarcoma* [Title/Abstract] or hematopoiet* sarcoma* [Title/Abstract] or blood sarcoma* [Title/Abstract] or "bone marrow" sarcoma*[Title/Abstract] or lymphoid sarcoma* [Title/Abstract] or "lymphoid tissue*" sarcoma*[Title/Abstract] or histiocytosis sarcoma* [Title/Abstract] or hematolog* tumor*[Title/Abstract] or hematopoiet* tumor* [Title/Abstract] or blood tumor* [Title/Abstract] or "bone marrow" tumor* [Title/Abstract] or lymphoid tumor* [Title/Abstract] or "lymphoid tissue*" tumor* [Title/Abstract] or histiocytosis tumor* [Title/Abstract] or hematolog* tumour* [Title/Abstract] or hematopoiet* tumour* [Title/Abstract] or blood tumour* [Title/Abstract] or "bone marrow" tumour* [Title/Abstract] or lymphoid tumour* [Title/Abstract] or "lymphoid tissue*" tumour* [Title/Abstract] or histiocytosis tumour* [Title/Abstract])) OR ((lymphoma* or "Germinoblastic Sarcoma*"[Title/Abstract] or Reticulolymphosarcoma* or Germinoblastoma* or Hodgkin* or "Non Hodgkin*"[Title/Abstract] or Non-Hodgkin* or NonHodgkin* or Lymphogranuloma* or "Immunoproliferative Small Intestinal Disease*"[Title/Abstract] or "alpha-Chain Disease*"[Title/Abstract] or "alpha chain disease*"[Title/Abstract] or "Small Cleaved Cell Lymphoma*"[Title/Abstract] or Reticulosarcoma* or "Burkitt* tumor*"[Title/Abstract] or "burkitt* tumour*"[Title/Abstract] or "Lymphomatoid Granulomatoses"[Title/Abstract] or "Lymphomatoid Granulomatosis"[Title/Abstract] or aleukaemia* or aleukemia* or hemoblastoma* or leukaemia* or leukemia* or leucaemia* or leucemia* or leukemogenesis or leucemia* or myeloma* or myeloplaxoma* or plasmacytoma* or "plasma cytoma*"[Title/Abstract] or "Kahler* disease"[Title/Abstract] or mastocytoma*)) AND (sarcopenia*[Title/Abstract] OR myopenia*[Title/Abstract] OR dynapenia*[Title/Abstract] OR frailty[Title/Abstract] OR frail[Title/Abstract] OR muscl* atroph*[Title/Abstract] OR muscl* wast*[Title/Abstract] OR muscl* loss*[Title/Abstract] OR muscl* mass*[Title/Abstract] OR muscl* function*[Title/Abstract] OR muscl* low[Title/Abstract] OR muscl* lowest[Title/Abstract] OR muscl* decreas*[Title/Abstract] OR muscl* strength*[Title/Abstract])

**Google Scholar**

hematologic malignancies sarcopenia

**
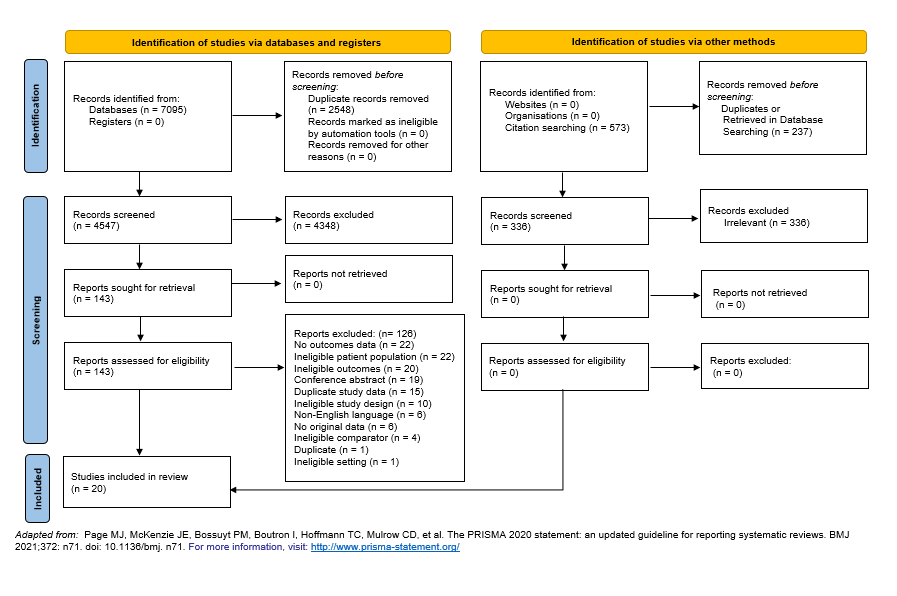
**

**Fig. 5** PRISMA flow sheet illustrating study selection process

**Supplementary Table 3: Excluded Studies Table**

| **First Authors Last Name** | **Year** | **Title** | **Journal** | **Reason for Exclusion** |
| --- | --- | --- | --- | --- |
| Amini | 2018 | Association of psoasmuscle uptake on FDG PET/Ct with progression from smoldering to symptomatic multiple myeloma | Skeletal Radiology | Ineligible patient population |
| Anderson | 2019 | Assessing Cachexia Acutely after Autologous Stem Cell Transplant | Cancers | Ineligible study design |
| Aoyama | 2017 | Nutritional risk in allogeneic stem cell transplantation: rationale for a tailored nutritional pathway | Annals of Hematology | Ineligible outcomes |
| Armenian | 2017 | Sarcopenia is a clinically relevant and independent predictor of adverse outcomes after allogeneic hematopoietic cell transplantation | Blood | Duplicate study data |
| Bas | 2020 | Relationship between sarcopenia and anthracycline related cardiotoxicity in patients with cancer | Annals of Oncology | Ineligible patient population |
| Beckerson | 2016 | Prevalence of sarcopenia prior to stem cell transplant | Clinical Nutrition | No outcome data |
| Bhamidipati | 2013 | Psoas cross-sectional area as radiographic measure of sarcopenia does not predict overall survival in multiple myeloma | Blood | Conference abstract |
| Bilen | 2018 | 133P Sarcopenia and inflammation predicts survival in advanced stage cancer patients (pts) treated with immunotherapy (IO) | Annals of Oncology | Ineligible patient population |
| Brown | 2019 | The evolution of body composition in oncology epidemiology, clinical trials, and the future of patient care: facts and numbers | Journal of Cachexia, Sarcopenia and Muscle | No original data |
| Brown | 2018 | The evolution of body composition in oncology- epidemiology, clinical trials, and the future of patient care: Facts and numbers | Journal of Cachexia, Sarcopenia and Muscle | No original data |
| Caram | 2015 | Sarcopenia is associated with autologous transplant-related outcomes in patients with lymphoma | Leukemia and Lymphoma | No outcome data |
| Caram | 2013 | The Impact of Sarcopenia on Transplant-Related Outcomes in Patients with Non-Hodgkin's and Hodgkin's Lymphoma | Abstracts/Biol Blood Marrow Transplant | Conference abstract |
| Caram | 2013 | The impact of sarcopenia on transplant-related complications and number of days spent in the hospital in patients with lymphoma | Journal of Clinical Oncology. Conference | Duplicate study data |
| Cencini | 2019 | Hodgkin lymphoma in the elderly: new perspectives and a 10-year monocenter real-life experience | Hematological Oncology | Ineligible patient population |
| Chu | 2013 | Indolent non-hodgkin lymphoma: Skeletal muscle density predicts overall survival with rituximab based chemotherapy | Blood | Conference abstract |
| Cintosun | 2015 | Understanding sarcopenia as a predictor of response to autologous hematopoietic stem cell transplant | Leukemia & Lymphoma | No original data |
| CostaNeto | 2018 | Association of Sarcopenia with Toxicities and Survival after Autologous Hematopoietic Stem Cell Transplantation for Adults with Lymphomas | Blood | Conference abstract |
| DaOrta | 2018 | Management and treatment of sarcopenia in fifty patients receiving chemotherapy with AHCC (active hexose correlated compound) | WORLD CANCER RESEARCH JOURNAL | Ineligible patient population |
| DeFilipp | 2018 | Evolution of Body Composition Following Autologous and Allogeneic Hematopoietic Cell Transplantation: Incidence of Sarcopenia and Association with Clinical Outcomes | Biology of Blood and Marrow Transplantation | Ineligible outcomes |
| Defilipp | 2017 | Longitudinal changes in body composition following hematopoietic cell transplantation | Blood | Ineligible outcomes |
| Deux | 2021 | High adipose tissue density is a negative prognostic factor in DLBCL patients treated by RCHOP, independent from tmtv and PS-from the remarc study | Hematological Oncology | Ineligible study design |
| Drory | 2020 | Sarcopenia and clinical evaluation in predicting treatment tolerability and outcomes in aggressive non-Hodgkin B cell lymphoma | Journal of Clinical Oncology | Conference abstract |
| Dubu | 2020 | Sarcopenia and diffuse large B cell lymphoma | Hematologie | Non-English language |
| Dunne | 2019 | Characterizing cancer cachexia in the geriatric oncology population | Journal of Geriatric Oncology | Ineligible patient population |
| Elfassy | 2019 | The Utility of Skeletal Muscle Cross Sectional Area and Nutritional Risk Index in Predicting Outcomes Across Patients with Hematologic Malignancy and Acute Respiratory Failure | American Journal of Respiratory and Critical Care Medicine | Conference abstract |
| Fukushima | 2019 | Characteristics of muscle function and the effect of cachexia in patients with haematological malignancy | European Journal of Cancer Care | No outcome data |
| Fukushima | 2020 | Factors associated with muscle function in patients with hematologic malignancies undergoing chemotherapy | Supportive Care in Cancer | No outcome data |
| Fukushima | 2019 | Influence of Hemoglobin Level on Muscle and Physical Functions, Activities of Daily Living, and Quality of Life in Patients With Hematological Malignancies | Integrative Cancer Therapies | Ineligible outcomes |
| Garrido |  | Detection and evaluation of the role of sarcopenia in elderly patients with cancer treated with chemotherapy. ONCOSARCO Project. Preliminary results | Rev Esp Geriatr Gerontol | Ineligible patient population |
| Gioia | 2017 | The Impact of Sarcopenia on Elderly Cancer Patients | Archive of Gerontology and Geriatrics Research | No original data |
| Gioia | 2017 | The Impact of Sarcopenia on Elderly Cancer Patients | Peertechz J Gerontol Geriatr Res | Ineligible study design |
| Go | 2019 | Clinical impact of prognostic nutritional index in diffuse large B cell lymphoma | Annals of Hematology | Duplicate study data |
| Go | 2017 | A comparison of pectoralis versus lumbar skeletal muscle indices for defining sarcopenia in diffuse large B-cell lymphoma - two are better than one | Oncotarget | Duplicate study data |
| Go | 2020 | Prognostic model based on the geriatric nutritional risk index and sarcopenia in patients with diffuse large B-cell lymphoma | BMC Cancer | Ineligible comparator |
| Go | 2016 | Prognostic significance of sarcopenia in patients with diffuse large B-cell lymphoma treated with rituximab plus CHOP (R-CHOP) | Haematologica | Conference abstract |
| Go | 2017 | Clinical relevance of sarcopenia in diffuse large B-cell lymphoma-two are better than one | Haematologica | Duplicate study data |
| Gourd | 2018 | Sarcopenia and adiposity linked to overall survival | The Lancet Oncology | Ineligible patient population |
| Greenfield | 2014 | Endocrine, metabolic, nutritional and body composition abnormalities are common in advanced intensively-treated (transplanted) multiple myeloma | Bone Marrow Transplantation | No outcome data |
| Guevara | 2020 | [Prognostic value of interim PET/CT in non-hodgkin lymphoma] | Revista Medica de Chile | Non-English language |
| Guo | 2021 | Body composition as a predictor of toxicity and prognosis in patients with diffuse large b-cell lymphoma receiving r-chop immunochemotherapy | Current Oncology | Ineligible patient population |
| Hilmi | 2019 | Body composition and sarcopenia: The next-generation of personalized oncology and pharmacology? | Pharmacology & Therapeutics | Ineligible study design |
| Hirota | 2020 | Risks of Sarcopenia in Patients with Hematological and Oncological Factors who Underwent Hematopoietic Stem Cell Transplantation | The Japanese Journal of Rehabilitation Medicine | Non-English language |
| Jabbour | 2019 | Sarcopenic obesity derived from PET/CT predicts mortality in lymphoma patients undergoing hematopoietic stem cell transplantation | Current Research in Translational Medicine | Ineligible outcomes |
| Kamiya | 2018 | A Prospective Observational Study Evaluating Sarcopenia By Using the Bioelectrical Impedance Analysis in Elderly Patients with Hematologic Malignancies | Blood | No outcome data |
| Karmali | 2017 | Impact of cachexia on outcomes in aggressive lymphomas | Annals of Hematology | Ineligible comparator |
| Karmali | 2016 | Biologic pathways of cancer-cachexia syndrome (Muscle and Fat Wasting) impact clinical outcomes in aggressive B-cell non-hodgkin lymphoma | Blood | Conference abstract |
| Khan | 2019 | Mri quantitation of abdominal skeletal muscle correlates with CT-based analysis: implications for sarcopenia measurement | Applied Physiology, Nutrition, and Metabolism | Ineligible outcomes |
| Kim | 2013 | Low psoas muscle/BMI ratio is a bad prognostic index of non-relapse survival after allogeneic stem cell transplantation | Blood | Conference abstract |
| Koch | 2016 | Neutrophil engraftment and graft-versus-host disease in elderly patients undergoing hematopoietic stem cell transplantation: Importance of body composition assessment and geriatric | Blood | Ineligible outcomes |
| Koyuncu | 2020 | Can sarcopenia be a risk factor for bleomycin toxicity? | Hematology, Transfusion and Cell Therapy | Ineligible outcomes |
| Kraut | 2011 | Sarcopenia determined by computed tomography (CT) imaging is a better prognosis factor than albuminemia or Charlson index in elderly patients with diffuse large B-cell lymphoma (DLBCL) | Blood | Conference abstract |
| Kurniawan | 2019 | Correlation between bio-impedance analysis and abdominal CT scan to diagnose decreased muscle mass in adult cancer patients | Annals of Oncology | Ineligible patient population |
| Lamisse | 1987 | Changes in nutritional status in the initial phase of treatment of cancers and malignant blood diseases. [French] | Revue de Medecine Interne | Non-English language |
| Lanic | 2012 | Sarcopenia determined by computed tomography imaging is an independant prognostic factor in elderly patients with diffuse large B CELL lymphoma treated by immunochemotherapy | Blood | Conference abstract |
| Lanic | 2013 | Clinical relevance of cachexia assessed by an anthropometric tool in elderly patients with diffuse large b-cell lymphoma treated by immunochemotherapy | Blood | Duplicate study data |
| Lee | 2018 | Clinical impact of prognostic nutritional index in diffuse large B-cell lymphoma | HemaSphere | Conference abstract |
| Ligibel | 2020 | Sarcopenia in aging, obesity, and cancer | Translational cancer research | No original data |
| Lin | 2019 | The Geriatric Syndrome of Sarcopenia Impacts Allogeneic Hematopoietic Cell Transplantation Outcomes in Combination with Multi-Morbidity and Functional Impairment | Leuk Lymphoma | Conference abstract |
| Liva | 2020 | Population pharmacokinetic (PK) analysis from first-in-human data for HDAC inhibitor, REC-2282, in patients with solid tumors and hematologic malignancies | Clinical Pharmacology and Therapeutics | Ineligible patient population |
| Loh | 2019 | Integrating Assessment of Sarcopenia into Decision-making for Allogeneic Hematopoietic Cell Transplantation: Ready for Prime Time? | Journal of the National Cancer Institute | No original data |
| Lucijanic | 2021 | Prognostic impact of psoas muscle index in patients with diffuse large B-cell lymphoma might be dependent on the immunochemotherapy type | Leukemia & Lymphoma | No outcome data |
| Lucijanic | 2021 | Psoas muscle index at the time of diagnosis might reflect the prognosis of classical Hodgkins lymphoma patients | Wiener Klinische Wochenschrift | No outcome data |
| Lucijanic | 2021 | Psoas muscle index at the time of diagnosis might reflect the prognosis of classical Hodgkin's lymphoma patients | Wiener Klinische Wochenschrift | Duplicate |
| Mallard | 2017 | Cachexia in lymphoma patients: Preliminary results of prevalence and risk factors | Journal of Cachexia, Sarcopenia and Muscle | No outcome data |
| Mallard | 2019 | C-Reactive Protein Level: A Key Predictive Marker of Cachexia in Lymphoma and Myeloma Patients | Journal of Hematology | Ineligible comparator |
| Mallard | 2019 | Preliminary results of the cachexia prevalence study in hematology from HEMODIAG cohort: Prevention by adapted physical activity perspectives | Movement and Sports Sciences | Non-English language |
| Mishra | 2020 | CT-Defined Fat Index Is a Prognostic Factor of Chronic Graft-Versus-Host Disease Outcomes in Adult Allogeneic Transplant Recipients | Biology of Blood and Marrow Transplantation | Duplicate study data |
| Mishra | 2019 | CT-Defined Body Composition in Adult Patients with Hematological Malignancies Prior to Allogeneic Hematopoietic Stem Cell Transplantation (alloHSCT): Endemic Sarcopenia and Reduced Muscle Radiodensity Associate with Impaired Forced Expiratory Volume (FEV) | Biol. Blood Marrow Transplant. | Ineligible outcomes |
| Mishra | 2020 | Sarcopenia and low muscle radiodensity associate with impaired FEV<inf>1</inf> in allogeneic haematopoietic stem cell transplant recipients | Journal of Cachexia, Sarcopenia and Muscle | Ineligible outcomes |
| Mishra | 2019 | CT-Defined Body Composition in Adult Patients with Hematological Malignancies Prior to Allogeneic Hematopoietic Stem Cell Transplantation (alloHSCT): Endemic Sarcopenia and Reduced Muscle Radiodensity Associate with Impaired Forced Expiratory Volume (FEV)1 | Biology of Blood and Marrow Transplantation | No outcome data |
| Morishita | 2012 | Prevalence of sarcopenia and relevance of body composition, physiological function, fatigue, and health-related quality of life in patients before allogeneic hematopoietic stem cell transplantation | Supportive Care in Cancer | No outcome data |
| Morishita | 2011 | The incidence of sarcopenia and its effects on body composition, physiological function, nutrition, and fatigue in patients before allogeneic hematopoietic stem cell transplantation | Journal of Cachexia, Sarcopenia and Muscle | No outcome data |
| Mueske | 2019 | Myosteatosis in adolescents and young adults treated for acute lymphoblastic leukemia | Leukemia and Lymphoma | Ineligible patient population |
| Nagata | 2019 | Weight adjusted urinary creatinine excretion predicts transplant outcomes in adult patients with acute myeloid leukemia in complete remission | Blood | Ineligible study design |
| Nakamura | 2018 | Prognostic impact of skeletal muscle assessed by computed tomography scan in patients with acute myeloid leukemia | HemaSphere | Duplicate study data |
| Nakamura | 2015 | Sarcopenia and adipopenia are independent prognostic factors in patients with acute myeloid leukemia | Annals of Oncology | Duplicate study data |
| Nakamura | 2018 | Alteration of muscle mass after chemotherapy in patients with newly diagnosed multiple myeloma | Annals of Oncology | No outcome data |
| Ninomiya | 2018 | Sarcopenia in patients with diffuse large B-cell lymphoma is caused by the deficiency of essential amino acid tryptophan | HemaSphere | No outcome data |
| Oflazoglu | 2020 | Prevalence and related factors of sarcopenia in newly diagnosed cancer patients | Supportive Care in Cancer | Ineligible outcomes |
| Paillaud | 2019 | Nutritional Status Assessment in Older Patients with Cancer: A National Cross-Sectional Survey (Nutriagecancer) | Clinical Nutrition | Ineligible patient population |
| Park | 2017 | Low pectoralis muscle mass is a good prognostic factor in patients with diffuse large B-cell lymphoma | Journal of Thoracic Imaging | Conference abstract |
| Pereira | 2017 | Pre-sarcopenia and bone mineral density in adults submitted to hematopoietic stem cell transplantation | Rev Bras Hematol Hemoter | Ineligible outcomes |
| PerezCamargo | 2017 | Assessment and Impact of Phase Angle and Sarcopenia in Palliative Cancer Patients | Nutrition and Cancer | Ineligible patient population |
| Persoon | 2017 | Health-related physical fitness in patients with multiple myeloma or lymphoma recently treated with autologous stem cell transplantation | Journal of Science and Medicine in Sport | Ineligible study design |
| Pires-Avancini | 2020 | RISK OF SARCOPENIA AND ASSOCIATION WITH NUTRITIONAL STATE AND HANDGRIP STRENGTH IN PATIENTS WITH HEMATOLOGICAL CANCER | Ann. Nutr. Metab. | No outcome data |
| Prica | 2019 | Functional predictors of chemotherapy toxicity in elderly lymphoma patients-a prospective pilot study | Hematological Oncology | Ineligible outcomes |
| Privitera | 1961 | [Myelogenic muscular atrophy in leukemic lymphadenosis] | Bollettino - Societa Medico Chirurgica Cremona | Non-English language |
| Ravasco | 2013 | PP101-SUN DIETARY INTAKE, SARCOPENIA AND PHASE ANGLE IN CANCER: NEW INSIGHTS | Clinical Nutrition | Ineligible patient population |
| Ren | 2017 | Protein blend ingestion before allogeneic stem cell transplantation improves protein-energy malnutrition in patients with leukemia | Nutrition Research | Ineligible patient population |
| Rezk | 2018 | Bioimpedance vector analysis for the detection of extracellular volume overload and sarcopenia in systemic AL amyloidosis | British Journal of Haematology | Ineligible patient population |
| Rier | 2019 | Prognostic Impact of Low Muscle Mass and Muscle Density in Patients with Diffuse Large B-Cell Lymphoma | Journal of Geriatric Oncology | Duplicate study data |
| Rier | 2018 | Severe sarcopenia might be associated with a decline of physical independence in older patients undergoing chemotherapeutic treatment | Supportive Care in Cancer | Ineligible outcomes |
| Rodriguez-Torres | 2019 | Sarcopenia in patients with malignant pleural effusion: impact on symptoms, health status, and response to hospitalization | Supportive Care in Cancer | Ineligible patient population |
| Sakatoku | 2016 | Prognostic impact of the subcutaneous adipose tissue loss on survival outcome in patients with multiple myeloma | Biology of Blood and Marrow Transplantation | Duplicate study data |
| Schink | 2018 | Whole-Body Electromyostimulation Combined With Individualized Nutritional Support Improves Body Composition in Patients With Hematological Malignancies - A Pilot Study | Frontiers in Physiology | Ineligible outcomes |
| Shibasaki | 2019 | Depletion of pre-transplant skeletal muscle is a significant poor prognostic factor in allogeneic hematopoietic cell transplantation | Blood | Conference abstract |
| Stege | 2018 | Geriatric impairments and low muscle mass are associated with treatment discontinuation and overall survival in newly diagnosed non-transplant eligible multiple myeloma patients (nte-NDMM) treated with dose-adjusted melphalan-prednisone-bortezomib (MPV) results of the Dutch HOVON 123 study | Blood | Conference abstract |
| Steinmeyer | 2019 | Low lean mass and chemotherapy toxicity risk in the elderly: The Fraction study protocol | BMC Cancer | Ineligible patient population |
| Sun | 2019 | Sarcopenia As a Significant Prognostic Factor in Acute Myeloid Leukemia: Validation of a Novel Scoring System | Blood | Conference abstract |
| Sun | 2015 | An Epidemiological Survey of Cachexia in Advanced Cancer Patients and Analysis on Its Diagnostic and Treatment Status | Nutrition and Cancer | No outcome data |
| Surov | 2010 | Intramuscular manifestation of non-Hodgkin lymphoma and myeloma: Prevalence, clinical signs, and computed tomography features | Acta Radiologica | No outcome data |
| Takekiyo | 2015 | Effect of exercise therapy on muscle mass and physical functioning in patients undergoing allogeneic hematopoietic stem cell transplantation | Supportive Care in Cancer | Ineligible study design |
| Tanaka | 2017 | Clinical impact of sarcopenia and relevance of nutritional intake in patients before and after allogeneic hematopoietic stem cell transplantation | Journal of Cancer Research and Clinical Oncology | Ineligible outcomes |
| Teranaka | 2017 | Clinical impact of sarcopenia and skeletal muscle mass change during chemotherapy on outcomes of diffuse large b-cell lymphoma | Blood | Conference abstract |
| Ueda | 2017 | Body composition assessment in hematopoietic stem cell transplantation | Revista Brasileira de Hematologia e Hemoterapia | Ineligible study design |
| Vaxman | 2016 | Muscle mass in elderly patients with DLBCL treated with RCHOP | Blood | Conference abstract |
| Viana | 2020 | Evaluation of nutritional risk factors in hematopoietic stem cell transplantation-eligible patients | Einstein | Ineligible outcomes |
| Wang | 2020 | Cancer-related fatigue and biochemical parameters among cancer patients with different stages of sarcopenia | Supportive Care in Cancer | No outcome data |
| Williams | 2019 | Sarcopenia & aging in cancer | Journal of Geriatric Oncology | Ineligible study design |
| Xiao | 2016 | Impact of sarcopenia on treatment tolerance in United States veterans with diffuse large B-cell lymphoma treated with CHOP-based chemotherapy | American Journal of Hematology | Ineligible outcomes |
| Yin | 2019 | Student presentation, encore presentation biochemical & physiologic factors that affect patient fitness & body composition during hematopoietic stem cell transplantation for hematologic malignancies | Journal of the American Geriatrics Society | Ineligible outcomes |
| Zakaria | 2020 | Sarcopenia Predicts Overall Survival in Patients with Lung, Breast, Prostate, or Myeloma Spine Metastases Undergoing Stereotactic Body Radiation Therapy (SBRT), Independent of Histology | Neurosurgery | Ineligible patient population |
| Zhang | 2016 | Incidence and risk factor analysis for sarcopenia in patients with cancer | Oncology Letters | No outcome data |
| Zhou | 2018 | Development and validation of a clinically applicable score to classify cachexia stages in advanced cancer patients | Journal of Cachexia, Sarcopenia and Muscle | Ineligible patient population |
| Zweegman | 2016 | Feasibility and efficacy of dose adjusted melphalan-prednisone-bortezomib (MPV) in elderly patients >= 75 years of age with newly diagnosed multiple myeloma; The non-randomised phase II HOVON 123 study | Blood | Ineligible patient population |
|  | 2020 | Impact of an APA Program on EFS in Patients With Diffuse Large-cell B Lymphoma Treated in 1st Line |  | Ineligible study design |
|  | 2010 | Women With Cancer: an Exercise Study to Promote Health |  | Ineligible outcomes |
|  | 2006 | A Randomized Trial of Human Growth Hormone (hGH) vs Placebo in Intensively Treated Haemato-Oncology Patients |  | Ineligible comparator |
|  | 2017 | Effects of evidence-based exercise on body composition, physical fitness, and recovery-related parameters in Hematopoietic Stem Cell Transplantation Patients |  | No outcome data |
|  | 2017 | Strength and Aerobic Training in Elderly Lymphoma Patients During Chemotherapy and Its Impact on Treatment Outcomes, Patients Functioning and Biological Markers of Aging |  | No outcome data |
|  | 2019 | Effects of Exercise on Health Outcomes in Multiple Myeloma |  | Ineligible setting |
|  | 2013 | Feasibility and Efficacy of dose adjusted Melphalan Prednisone Bortezomib (MPV) in elderly patients = 75 years of age with newly diagnosed Multiple Myeloma; a nonrandomised phase II study |  | No outcome data |


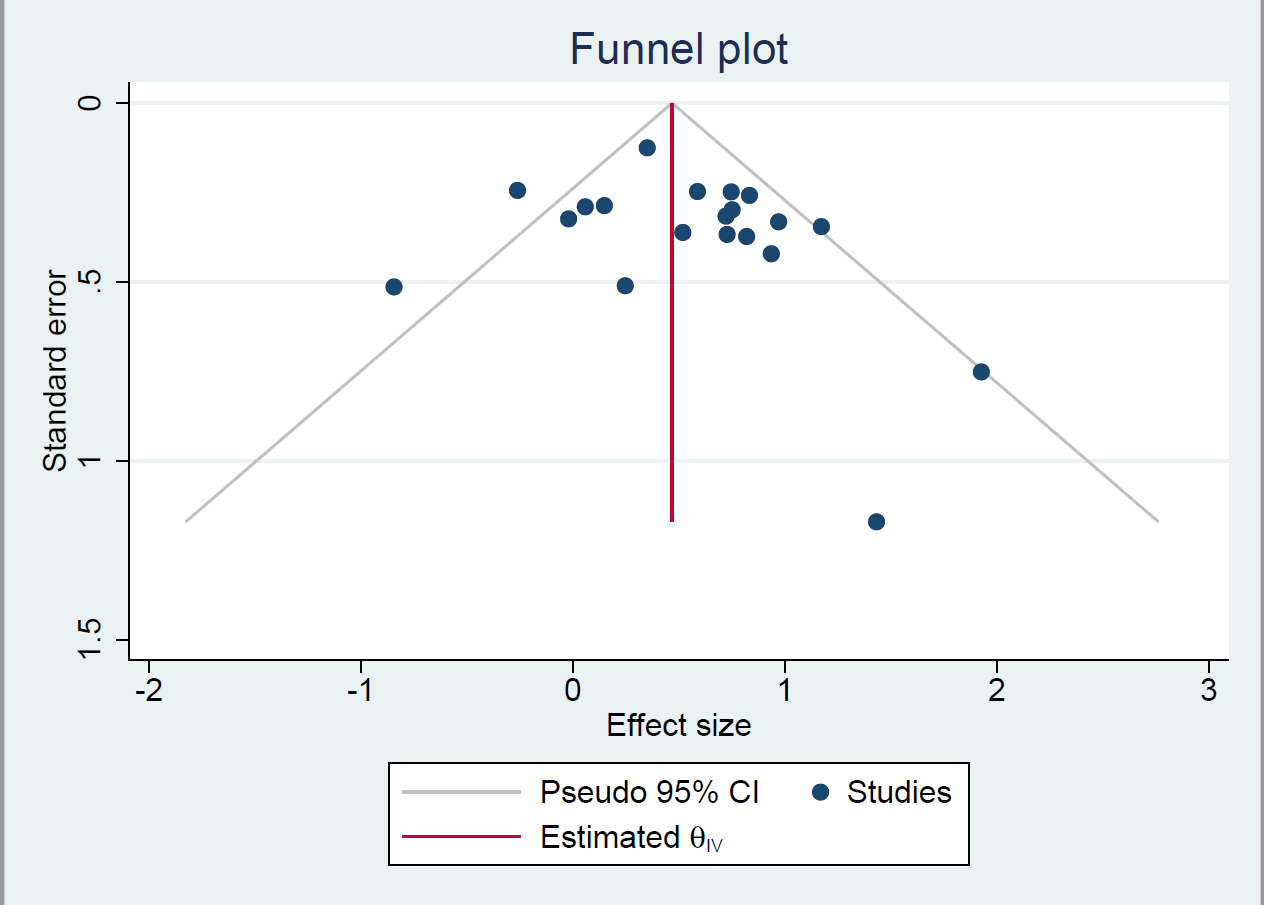


**Fig. 6** Funnel plot for graphical evaluation of publication bias. Study-level logarithmically transformed hazards ratios for overall survival are plotted in the x-axis, whereas standard error of this estimate (measure of precision) is plotted in the y-axis. A symmetric appearing inverted funnel plot generally suggests that publication bias is unlikely


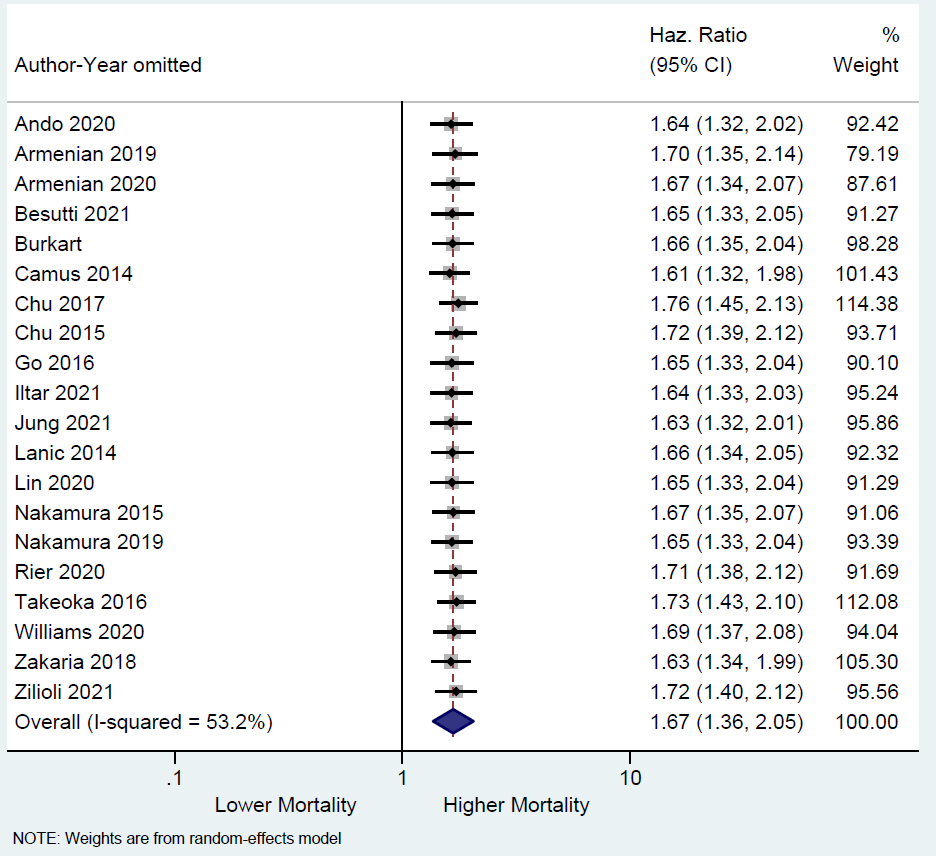


**Fig. 7** Results of the leave-one-out meta-analysis to detect influential/outlier studies. The relatively stability of hazard ratio, after excluding all the studies one by one, suggest that there are no outliers


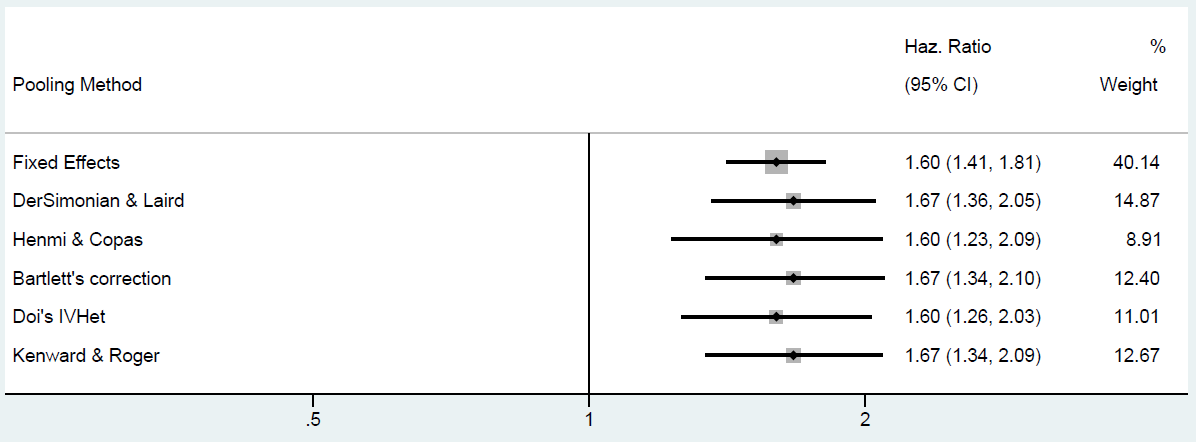


**Fig. 8** Pooled effect size for overall survival using alternative pooling strategies. The pooled hazard ratios remain relatively consistent regardless of the pooling method


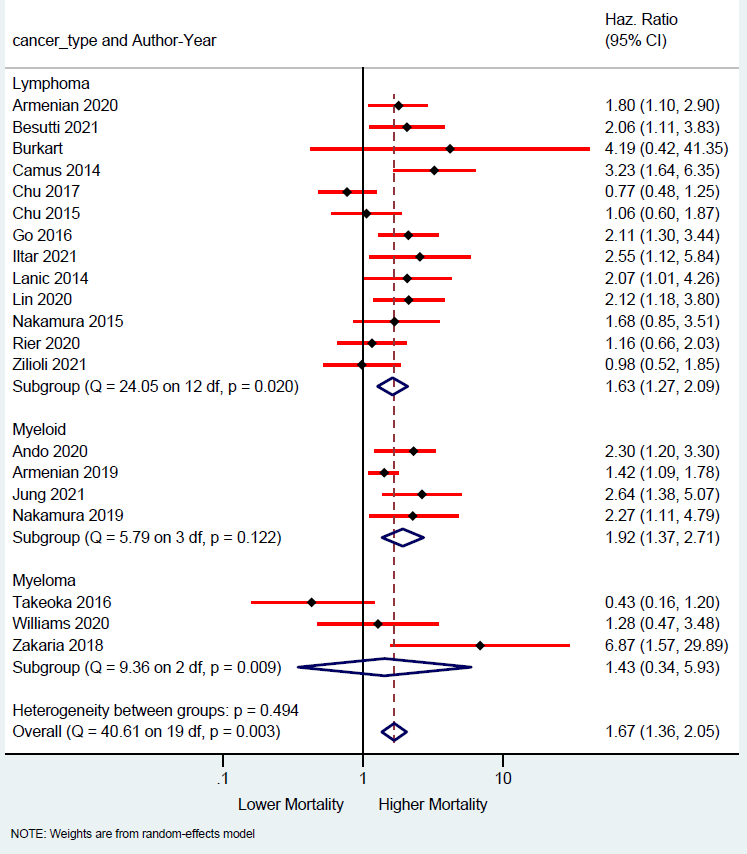


Cancer Type and Author (Year)

**Fig. 9** Subgroup analysis of the impact of Low Skeletal Muscle Mass based on malignancy subtype. The effect size point estimates remained relatively consistent regardless of cancer type, although technically not statistically significant among patients with multiple myeloma
